# The Effect of Resistance Exercise on Inflammatory and Myogenic Markers in Patients with Chronic Kidney Disease

**DOI:** 10.3389/fphys.2017.00541

**Published:** 2017-07-28

**Authors:** Emma L. Watson, Joao L. Viana, David Wimbury, Naomi Martin, Neil J. Greening, Jonathan Barratt, Alice C. Smith

**Affiliations:** ^1^Infection, Immunity and Inflammation, University of Leicester Leicester, United Kingdom; ^2^Research Center in Sports Sciences, Health Sciences and Human Development, CIDESD, University Institute of Maia, ISMAI Porto, Portugal; ^3^School of Sport, Exercise and Health Sciences, Loughborough University Loughborough, United Kingdom; ^4^Department of Respiratory Medicine, Institute for Lung Health, University Hospitals of Leicester NHS Trust Leicester, United Kingdom; ^5^John Walls Renal Unit, University Hospitals of Leicester NHS Trust Leicester, United Kingdom

**Keywords:** resistance exercise, CKD, cachexia, protein synthesis, protein degradation, inflammation, myogenesis

## Abstract

**Background:** Muscle wasting is a common complication of Chronic Kidney Disease (CKD) and is clinically important given its strong association with morbidity and mortality in many other chronic conditions. Exercise provides physiological benefits for CKD patients, however the molecular response to exercise remains to be fully determined. We investigated the inflammatory and molecular response to resistance exercise before and after training in these patients.

**Methods:** This is a secondary analysis of a randomized trial that investigated the effect of 8 week progressive resistance training on muscle mass and strength compared to non-exercising controls. A sub-set of the cohort consented to vastus lateralis skeletal muscle biopsies (*n* = 10 exercise, *n* = 7 control) in which the inflammatory response (IL-6, IL-15, MCP-1 TNF-α), myogenic (MyoD, myogenin, myostatin), anabolic (P-Akt, P-eEf2) and catabolic events (MuRF-1, MAFbx, 14 kDa, ubiquitin conjugates) and overall levels of oxidative stress have been studied.

**Results:** A large inflammatory response to unaccustomed exercise was seen with IL-6, MCP-1, and TNF-α all significantly elevated from baseline by 53-fold (*P* < 0.001), 25-fold (*P* < 0.001), and 4-fold (*P* < 0.001), respectively. This response was reduced following training with IL-6, MCP-1, and TNF-α elevated non-significantly by 2-fold (*P* = 0.46), 2.4-fold (*P* = 0.19), and 2.5-fold (*P* = 0.06), respectively. In the untrained condition, an acute bout of resistance exercise did not result in increased phosphorylation of Akt (*P* = 0.84), but this was restored following training (*P* = 0.01). Neither unaccustomed nor accustomed exercise resulted in a change in myogenin or MyoD mRNA expression (*P* = 0.88, *P* = 0.90, respectively). There was no evidence that resistance exercise training created a prolonged oxidative stress response within the muscle, or increased catabolism.

**Conclusions:** Unaccustomed exercise creates a large inflammatory response within the muscle, which is no longer present following a period of training. This indicates that resistance exercise does not provoke a detrimental on-going inflammatory response within the muscle.

## Introduction

Patients with Chronic Kidney Disease (CKD) commonly experience muscle wasting which can start early in the disease process not only impacting upon their quality of life and physical functioning, but there is also evidence that it increases their risk of death (Carrero et al., 2008; Isoyama et al., 2014; Harada et al., 2017) as in other chronic diseases (Zhou et al., 2010; Greening et al., 2015). A reduced muscle mass was found to be associated with mortality risk in end stage renal patients under conservative care (Pereira et al., 2015), but in patients undergoing hemodialysis, reduced strength was found to have a stronger association with risk of death rather than a low muscle mass (Isoyama et al., 2014). Evidence is lacking if this association is also true for patients at earlier stages of CKD. Recently physical function has been shown to be associated with mortality in both pre dialysis as well as dialysis patients (Morishita et al., 2017). The cause of this muscle loss is not yet fully understood, but is likely to be multifactorial including metabolic acidosis, inflammation, insulin resistance, oxidative stress, and physical inactivity (Mak and Cheung, 2006; Johansen and Painter, 2012).

There is a growing body of evidence demonstrating exercise provides a multitude of benefits for CKD patients including improved physical functioning and exercise tolerance, increased muscle mass, reduced cardiovascular risk and systemic inflammation and improved quality of life, (Castaneda et al., 2001, 2004; Moinuddin and Leehey, 2008; Mustata et al., 2011; Kosmadakis et al., 2012; Baria et al., 2014; Viana et al., 2014; Greenwood et al., 2015) and which is discussed in more depth a recent review (Gould et al., 2014). For these reasons exercise is increasingly recommended as an important adjunct to usual care in CKD (Koufaki et al., 2015). However, the mechanism by which exercise creates an increase muscle mass, or the molecular responses mounted to a bout of exercise by CKD patients are not yet fully described. It is important to understand how exercise interacts with the mechanisms underlying muscle loss in CKD to inform future guidelines for optimal exercise prescription in this population.

We have shown that 8 weeks of resistance exercise training improved muscular strength and size (Watson et al., 2014). The aim of the work we report here was to investigate the molecular response this exercise elicited. Specifically we investigated the inflammatory response (IL-6, IL-15, MCP-1 TNF-α), myogenic (MyoD, myogenin, myostatin), anabolic (Akt, eEf2) and catabolic events (MuRF-1, MAFbx, 14 kDa, ubiquitin conjugates) and overall levels of oxidative stress to an acute bout of resistance exercise before and after 8 weeks of training. It was hypothesized that CKD patients would only exhibit a small molecular response to unaccustomed resistance exercise, which would be significantly increased with training.

## Materials and methods

**Figure 1:**
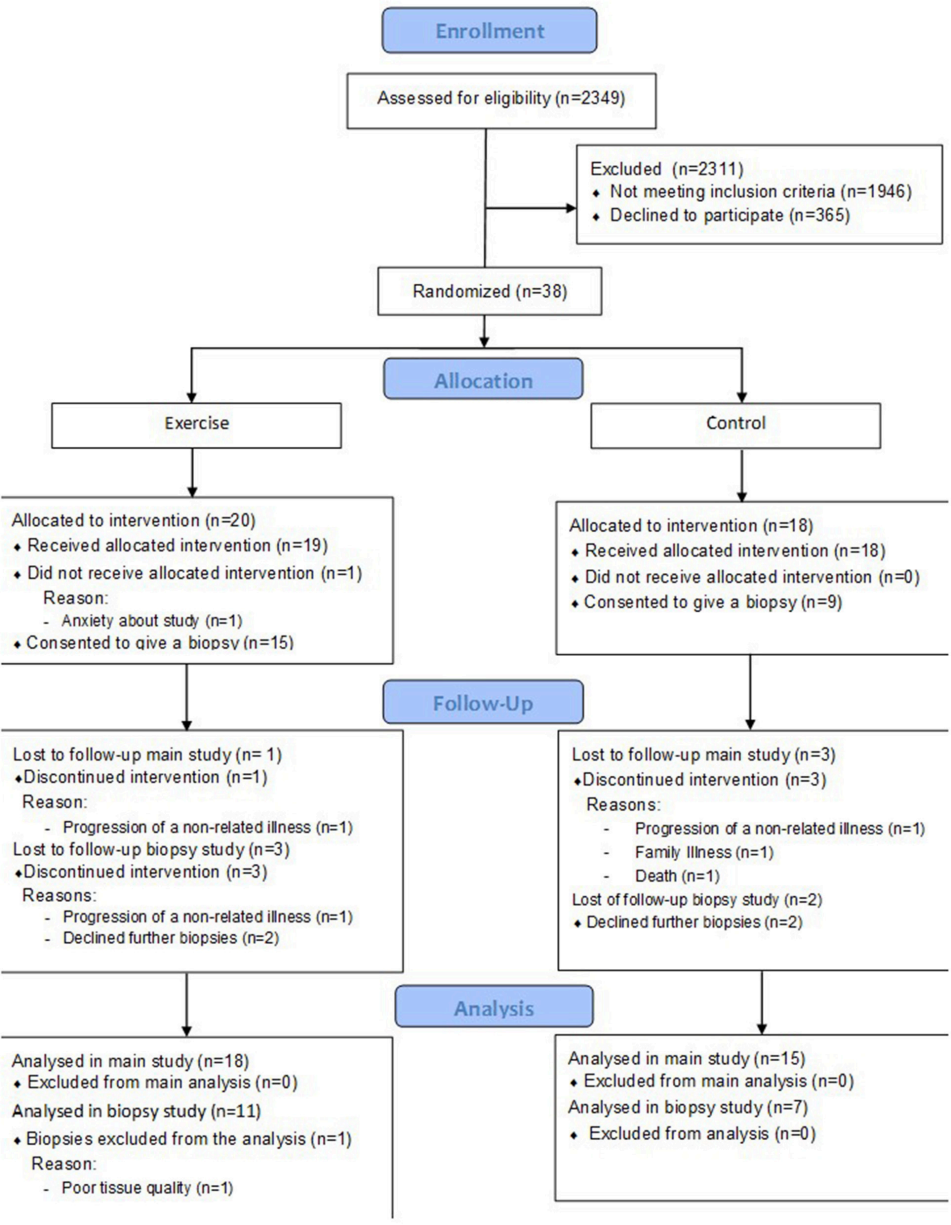
CONSORT diagram to show flow of patients through the study.

This is a secondary analysis of a randomized controlled trial described previously (Watson et al., 2014). Briefly, 38 patients with CKD stage 3b-4 were randomized to receive an 8 week resistance exercise training intervention (*n* = 20), or to the control group (*n* = 18). A sub-set of this main cohort also consented to skeletal muscle biopsies from vastus lateralis (exercise *n* = 11 however one patient was excluded due to poor tissue quality, analysis was performed on *n* = 10; control *n* = 7), the analysis of which is presented here. A CONSORT diagram describing the cohort is shown in Figure 1.

### Patients

Patient characteristics of this sub-cohort are presented in Table 1. All patients were recruited from nephrology outpatient clinics at Leicester General Hospital, UK. Patients were excluded from biopsy if receiving warfarin or clopidogrel, or suffering any clotting disorder. The study received approval from the UK National Research Ethics Committee, East-Midlands-Leicester (Ref 10/H0406/50); all patients gave written informed consent to participate in accordance with the Declaration of Helsinki.

**Table 1:** Patient characteristics.

**Characteristic**	**Exercise group (*n* = 11)**	**Control group (*n* = 7)**	***P-*value**
Age (yr)	62 ± 5	67 ± 11	0.17
Sex (*n* men/women)	6/5	6/1	0.05
Weight (kg)	97 ± 26	84 ± 18	0.24
Height (cm)	165 ± 12	165 ± 5	0.9
BMI (kg/m^2^)	36 ± 9	31 ± 6	0.19
eGFR (ml/min/1.73 m^2^)	27 ± 7	20 ± 6	0.09
Venous Bicarbonate (mmol/L)	26 ± 4	24 ± 2	0.72
Rectus Femoris CSA (cm^2^)	6.4 ± 2.0	5.1 ± 1.1	0.19
Isokinetic Strength (Nm)	109.3 ± 35.3	102.5 ± 46.9	0.52
Diabetes (%)	27	28	0.9

*Data are mean ± SD. BMI, body mass index; eGFR, estimated glomerular filtration rate (calculated using the 4-variavle MDRD [modification of Diet in Renal Disease] equation); CSA, cross sectional area*.

### Resistance exercise training

Patients randomized to the exercise group attended the hospital three times a week to participate in an 8 week progressive resistance training programme. Briefly, patients performed 3 sets of 10–12 repetitions of leg extension exercise at 70% 1-repetition maximum. When subjects could comfortably complete 3 sets with good form, the training load was increased. The control group was instructed to continue with their usual physical activity.

### Muscle biopsy sampling and processing

Three muscle biopsies were collected from the vastus lateralis of exercising patients at the following time points: Baseline, 24 h after the first exercise session (investigating the acute effect of exercise in the untrained state; untrained), 24 h after the last exercise session (investigating the acute effect of exercise in the trained state; trained). The timing of the biopsies was based on those employed in a similar study in patients with COPD (Constantin et al., 2013) that also investigated the molecular response to resistance exercise. Two muscle biopsies were collected from the control group, at baseline and 8 weeks later. All biopsies were taken after an overnight fast using the micro biopsy technique (Hayot et al., 2005). The site was cleaned using an iodine based solution and 5 ml 1% lidocaine administered. Samples were taken using an 11 g ACECUT automatic biopsy needle (TSK Laboratory, Netherlands) which yielded ~60 mg tissue. Samples were immediately placed in liquid nitrogen after dissection of any visible fat and connective tissue and freeze-dried (Edwards, Modulyo, UK with RV8 vacuum pump).

### Western blotting

Lysates were prepared by homogenization of ~5 mg/dw muscle tissue in Tris buffer containing 0.5 M EDTA, 40 nM EGTA, 10% Triton X-100, 0.1% betamercaptoethanol, supplemented with Phosphatase Inhibitor-3 (Sigma Aldrich, UK) and the following protease inhibitors Leupeptin (1 μg/ml), Pepstatin A (1 μg/ml), Benzamidine (1 mM) and PMSF (0.2 mM). Muscle extracts were rotated at 4°C for 90 min and centrifuged at 13,000 g for 15 min. Supernatant was collected and protein concentration determined using the Bio-Rad Protein Assay. Pellets were retained for determination of 14 kDa actin fragment (Du et al., 2004; Workeneh et al., 2006). Lysates containing 30 μg protein were subjected to SDS-PAGE using 10–12% gels on a mini-Protean Tetra system (Bio-Rad, UK). Proteins were transferred onto nitrocellulose membranes, blocked for 1 h with Tris-buffered saline with 5% (w/v) skimmed milk and 0.1% (v/v) Tween-20 detergent. Membranes were incubated with the primary antibody overnight. Antibodies to determine p-Akt (Ser^473^), p-eEF2 (Thr^56^) were obtained from Cell Signaling Technology (Danvers, MA, USA) 1:1,000 dilution, ubiquitin conjugates 1:150 dilution (Enzo Life Sciences, MI, USA) and AC40 Actin clone (Sigma Aldrich, UK) 1:500 dilution for analysis of 14 kDa actin fragment. The antibody against 14 kDa actin fragment also recognizes the 42 kDa fragment which was used as a loading control developed with a much shorter exposure time so not to be overexposed. For all other proteins β-actin (Abcam, Cambridge, UK) 1:5,000 dilution was used as a loading control. This was found to be the most stable of those tested (β-actin, α-tubulin and GAPDH). Oxidative stress was determined using the oxyblot protein oxidation detection kit (Merk Millipore, US). Bands were quantified using a Bio-Rad GS7000 densitometer and Molecular Analyst v1.4 Software. Due to the sample processing procedure for protein carbonylation analaysis, it was not possible to re-probe membranes for a loading control. To correct for protein concentration, lysates were also run for β-actin using the same protein calculations.

### Quantitative RT-PCR

RNA was isolated from 2 mg/dw muscle samples using TRIzol® (Invitrogen, UK) and reverse transcribed to cDNA using an AMV reverse transcription system (Promega, Madison, WI, USA). Primers, probes and internal controls for all genes were supplied as Taqman gene expression assays (Applied Biosystems, Warrington, UK. MAFbx:Hs00369714_m1, MuRF-1:Hs00822397_m1, Myogenin:Hs01072232_m1, MyoD:Hs02330075_g1, IL-6:Hs00985639_m1, IL-15:Hs01003716_m1, MCP-1:Hs00234140, TNF-α:Hs01113624_g1 and TBP:Hs00427620_m1 as an internal control which was determined to be stable over the course of the intervention (validation data is provided in Supplementary Methods section). All reactions were carried out in a 20 μl volume, 1 μl cDNA, 10 μl 2X Taqman Mastermix, 8 μl water, 1 μl primer/probe on an Agilent Biosystem Light Cycler with the following conditions, 95°C 15 s, followed by 40X at 95°C for 15 s and 60°C for 1 min. The Ct values from the target gene were normalized to TBP and expression levels calculated according to 2^−ΔΔCt^ method to determine fold changes. Data in graphs are presented as 2^−ΔCt^.

### Statistics

Data are presented as mean ± *SD* unless otherwise stated. All data sets were tested for normal distribution using the Kolmogorov–Smirnov test. For data shown to be skewed, log transformation was performed prior to analysis. Data was analyzed using repeated measures mixed ANOVA with pairwise comparisons of pre-specified comparisons of interest (baseline vs. untrained, baseline vs. trained in the exercise group and baseline vs. 8 weeks in the control group). This analysis was fitted using the xtmixed command in Stata v14. Statistical significance was accepted at *P* < 0.05.

## Results

### Intramuscular inflammatory and oxidative stress response to exercise

**Figure 2:**
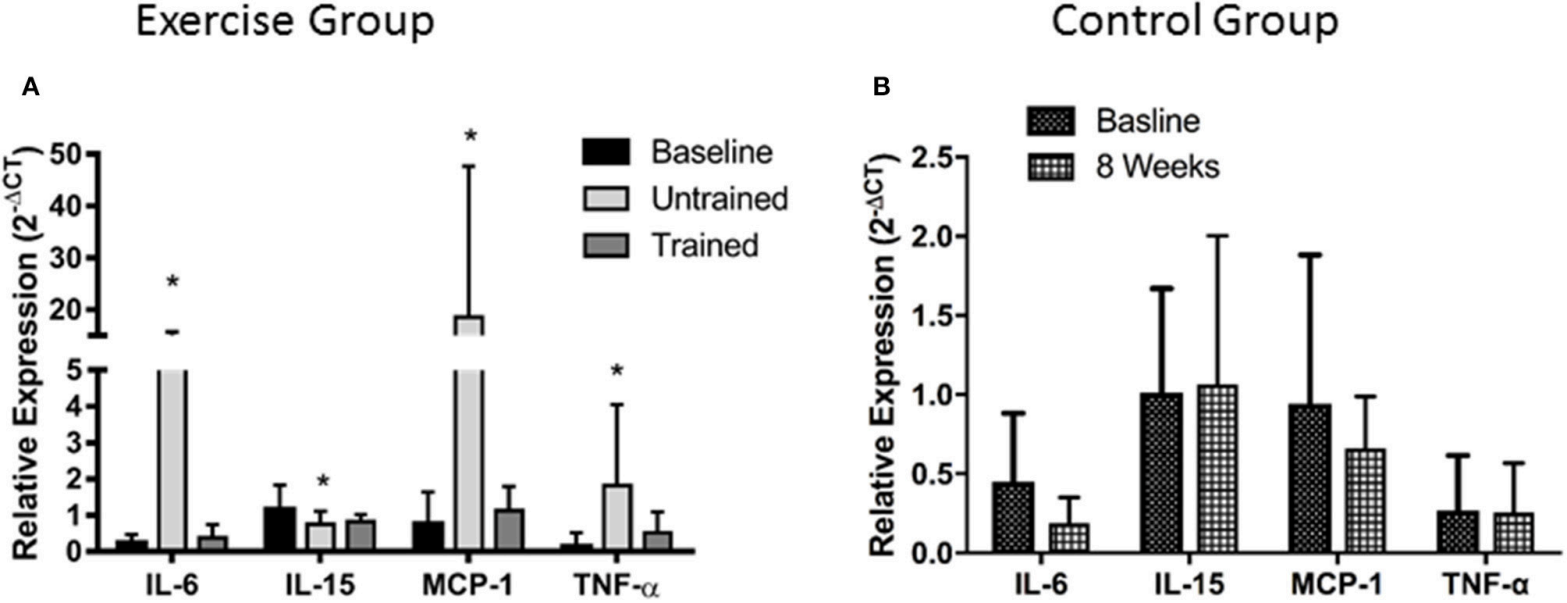
Changes in mRNA expression of intramuscular inflammatory cytokines after accustomed and unaccustomed resistance exercise. Skeletal muscle biopsies were drawn from exercising CKD patients at baseline, 24 h after first bout of exercise (Untrained) and 24 h after the final bout of exercise following 8 weeks of resistance exercise training (Trained) **(A)** and at baseline and after 8 weeks in the non-exercising control group **(B)**. ^*^P < 0.05 vs. representative baseline sample. Expression is displayed as relative change from baseline according to 2^−ΔCt^ method and normalized to Tata box binding protein. Data are mean ± *SD*.

**Figure 3:**
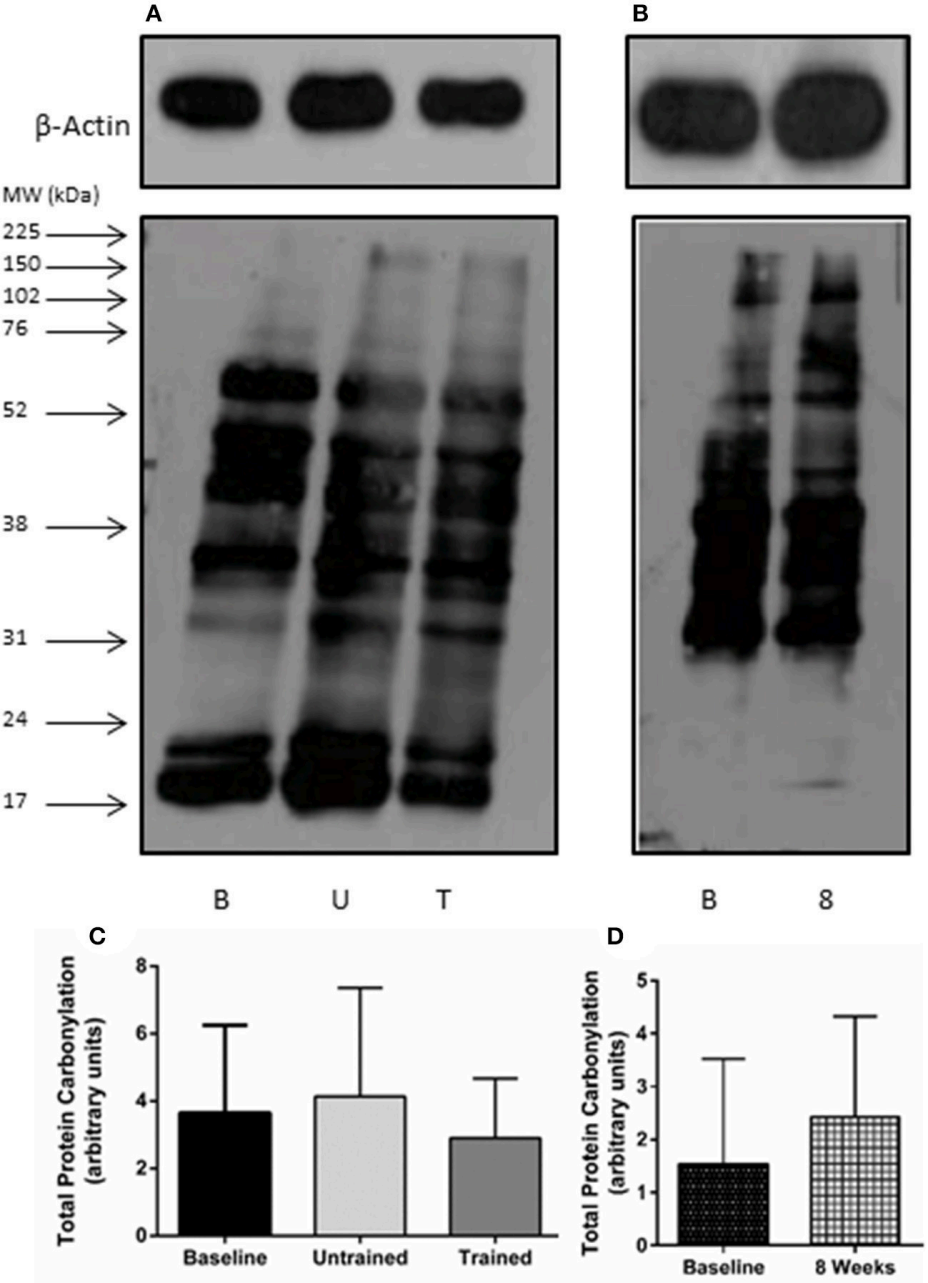
Total protein carbonylation as a marker of oxidative stress after accustomed and unaccustomed resistance exercise. Skeletal muscle biopsies were drawn from exercising CKD patients at baseline, 24 h after first bout of exercise (Untrained) and 24 h after the final bout of exercise following 8 weeks of resistance exercise training (Trained) and at baseline and after 8 weeks in the non-exercising control group. Representative oxyblots are shown for exercising **(A)** and control patients **(B)** together with β-actin loading controls for these groups. Histograms show densitometric data **(C,D)**. B, denotes Baseline; U, denotes untrained; T, denotes Trained; 8, denotes 8 weeks. Data are mean ± *SD*.

Unaccustomed resistance exercise induced a large increase in the expression of a number of inflammatory cytokines within skeletal muscle (Figure 2). 24 h after the first resistance exercise training session IL-6, MCP-1 and TNF-α mRNA expression were all up-regulated from baseline by means of 53-fold (*P* < 0.001), 25-fold (*P* < 0.001) and 4-fold (*P* < 0.001), respectively. These increases were blunted after 8 weeks of training with IL-6 increased just 2-fold when compared to baseline (*P* = 0.46), and MCP-1 2.4-fold (*P* = 0.19). There was a trend for TNF-α to still be elevated 2.5-fold above baseline in response to exercise following 8 weeks of training, but this fell short of significance (*P* = 0.06). IL-15 mRNA expression was significantly suppressed from baseline 24 h following the first bout of unaccustomed resistance exercise (*P* < 0.001), which was not seen following training (*P* = 0.46). Finally, acute exercise before or after training had no effect upon total protein carbonylation (Figure 3; *P* = 0.34), suggesting there was not a significant increase in oxidative stress in response to resistance exercise in these patients. Pairwise comparisons showed there was no change in the expression of IL-6, MCP-1, TNF-α, or IL-15 over the control period (*P* = 0.33, *P* = 0.76, *P* = 0.47, and *P* = 0.98, respectively).

### Changes in protein expression relating to myogenesis

**Figure 4:**
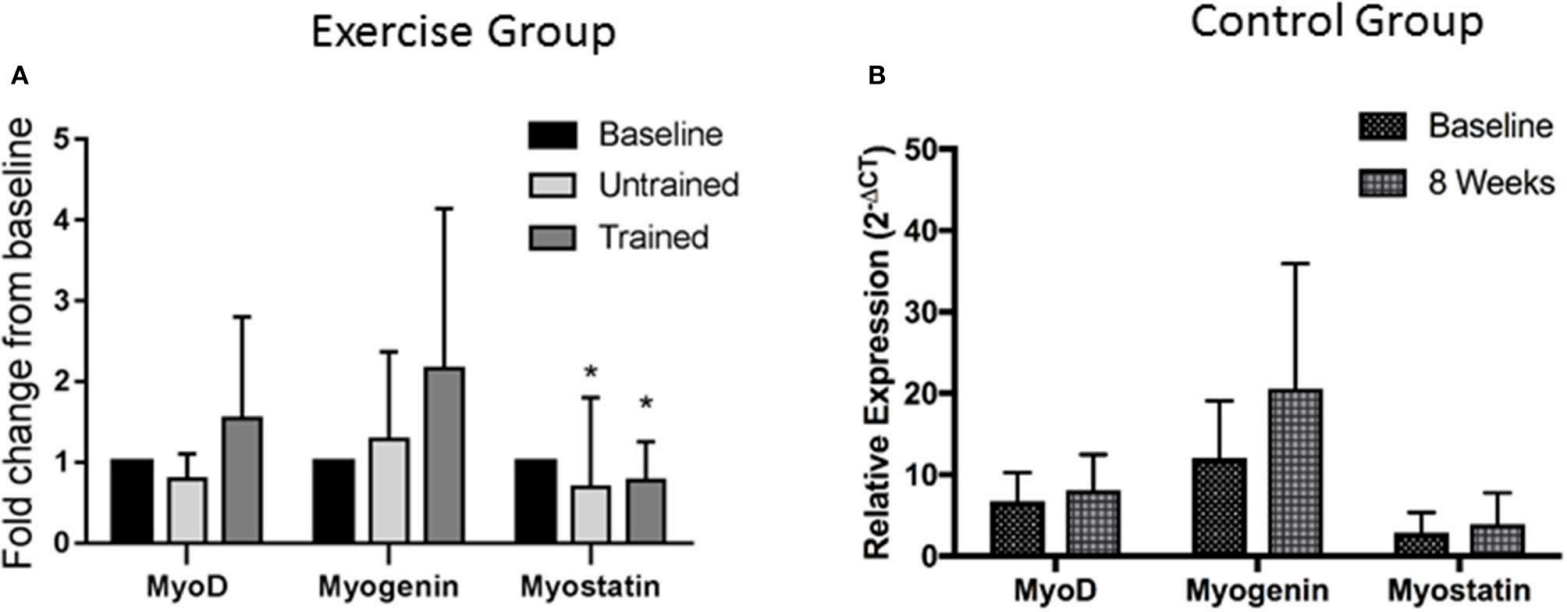
Changes in mRNA expression of proteins relating to Myogenesis after accustomed and unaccustomed resistance exercise. Skeletal muscle biopsies were drawn from exercising CKD patients at baseline, 24 h after first bout of exercise (Untrained) and 24 h after final bout of exercise following 8 weeks of resistance exercise training (Trained) **(A)** and at baseline and after 8 weeks in the non-exercising control group **(B)**. Expression is displayed as relative change from baseline according to 2^−ΔCt^ method and normalized to Tata box binding protein. ^*^P < 0.05 vs. respective baseline sample. Data are mean ± *SD*.

Changes in expression of genes relating to myogenesis are shown in Figure 4. Repeated measures mixed ANOVA revealed no change in MyoD (*P* = 0.90) or Myogenin (*P* = 0.88) mRNA expression following exercise either before or after training, suggesting that resistance exercise does not alter mRNA expression of these myogenic regulatory factors either positively or negatively before or after training at the time points assessed here. However, myostatin mRNA expression was significantly suppressed from baseline following exercise both prior to (*P* = 0.005) and following training (*P* = 0.04). Pairwise comparisons showed no change in mRNA expression of either MyoD (*P* = 0.18), Myogenin (*P* = 0.08) or myostatin (*P* = 0.78) in the control group over the 8 week period.

### Changes in proteins relating to protein synthesis

**Figure 5:**
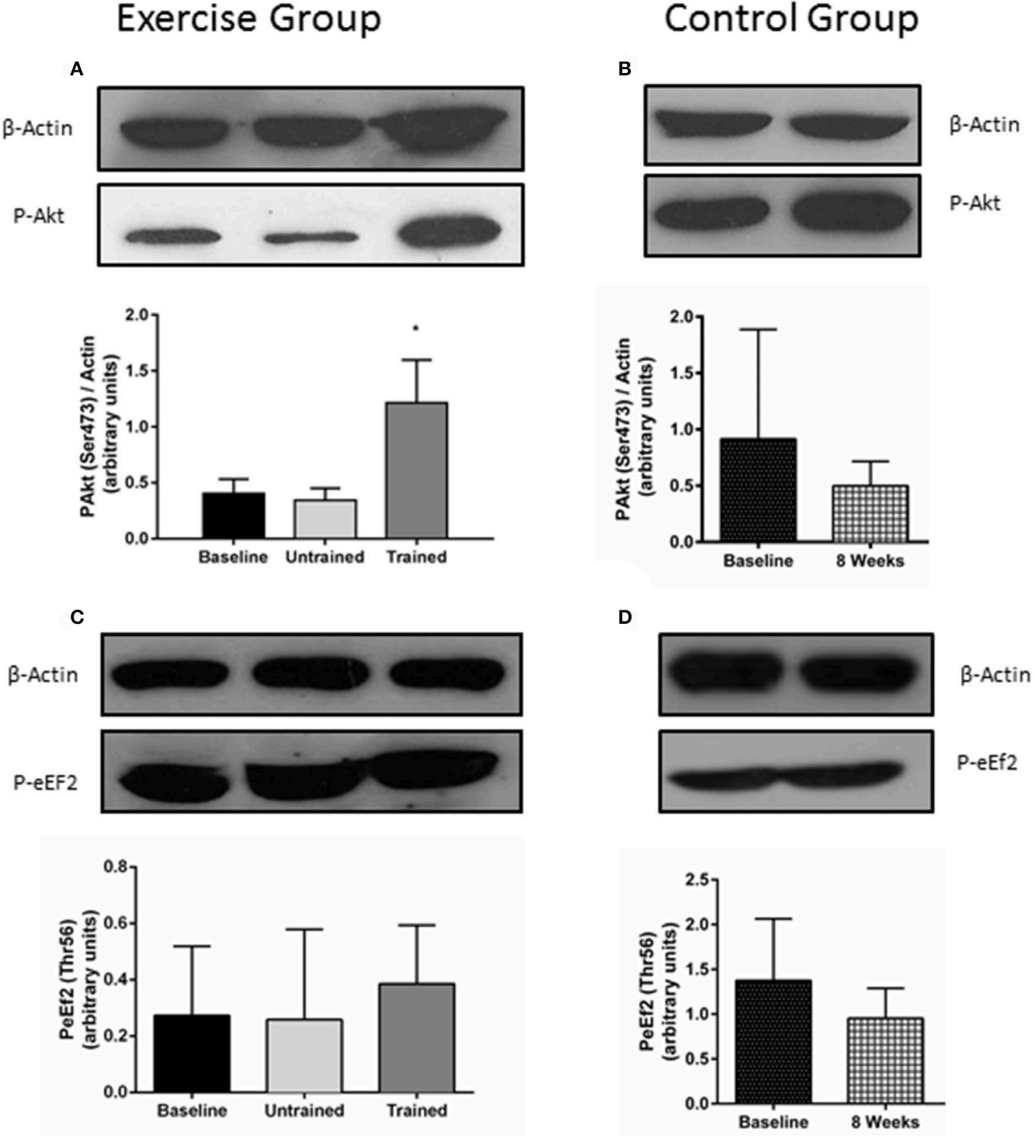
Changes in the phosphorylation status of proteins involved in protein synthesis in response to accustomed and unaccustomed resistance exercise. Skeletal muscle biopsies from exercising CKD patients were drawn at baseline, 24 h after first bout of exercise (Untrained) and 24 h after final bout of exercise following 8 weeks of resistance exercise training (Trained) **(A,C)** and at baseline and 8 weeks later in the non-exercising control group **(B,D)**. Phosphorylation levels of Akt on Ser473 and phosphorylation levels of eEF2 on Thr56 are normalized to β-Actin. Histograms show densitometric data and are shown with representative blots. ^*^P < 0.05 vs. respective baseline sample. Data are mean ± *SD*.

Changes in phosphorylation of Akt and eEf2 are shown in Figure 5. Akt phosphorylation increases in the hours following resistance exercise, although its necessity for muscle hypertrophy is in question. (Philp et al., 2011) There was no increase in Akt phosphorylation from baseline in response to acute unaccustomed exercise (*P* = 0.84). However, this response was somewhat restored after 8 weeks of resistance exercise training, where phosphorylation was seen to increase 2-fold above baseline (*P* = 0.01). Pairwise comparisons showed no change in the phosphorylation of Akt seen in the control group (*P* = 0.35). Repeated measures mixed ANOVA showed no effect of resistance exercise either before or after training on phosphorylation status of eEF2 (*P* = 0.44; Figure 5C).

### Changes relating to markers of protein degradation

**Figure 6:**
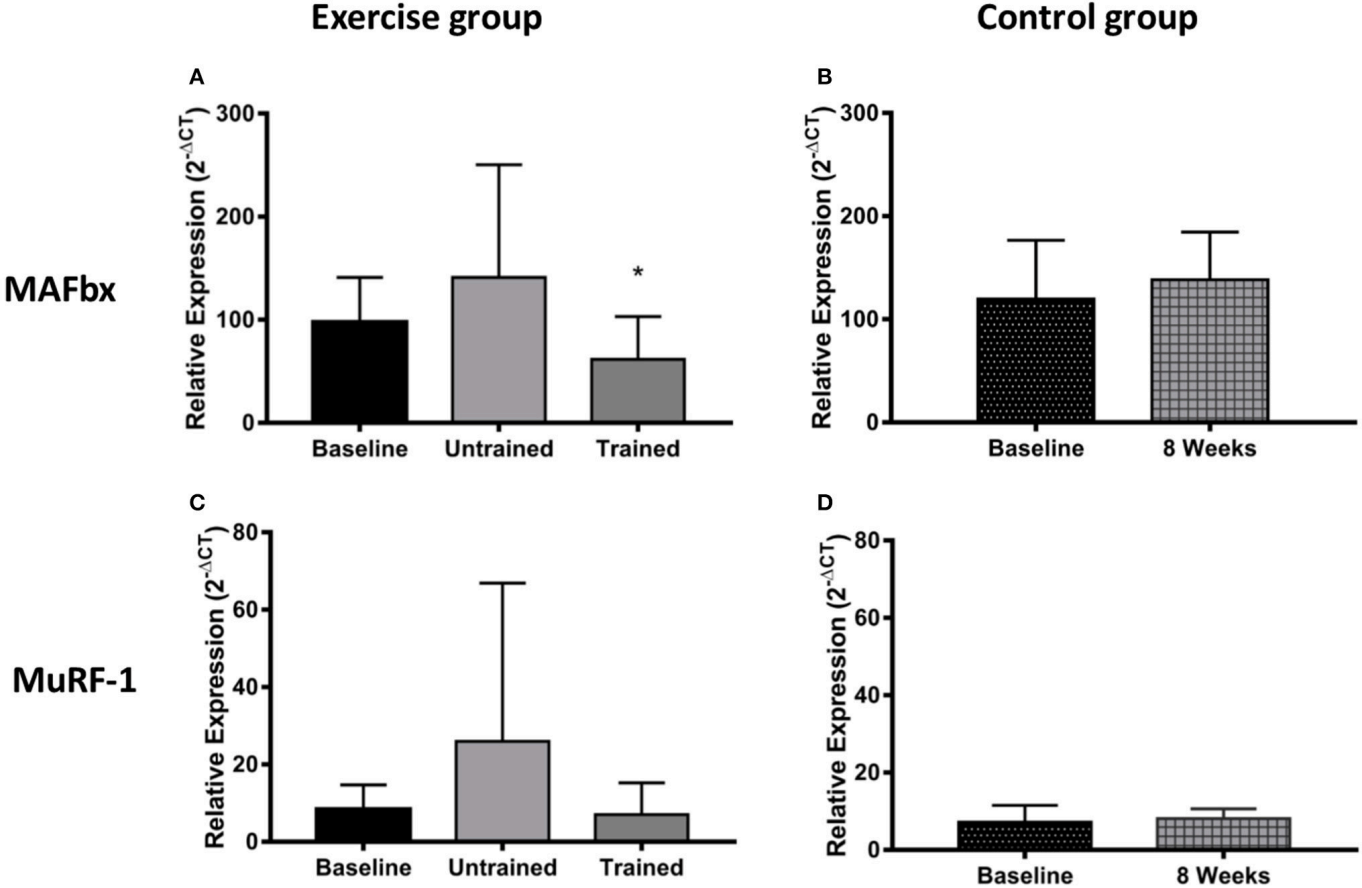
Changes in mRNA expression of proteins relating to muscle protein breakdown after accustomed and unaccustomed resistance exercise. Samples from exercising CKD patients were drawn at baseline, 24 h after the first exercise bout (Untrained) and 24 h after the final bout of exercise after 8 weeks of resistance exercise training (Trained) **(A,C)**. Samples from the non-exercising control group were drawn at baseline and 8 weeks later **(B,D)**. ^*^P < 0.05 vs. respective baseline sample. Expression is displayed as relative change from baseline according to 2^−ΔCt^ method and normalized to Tata box binding protein. Data are mean ± *SD*.

Changes in expression of genes relating to protein degradation are shown in Figure 6. An unaccustomed acute bout of resistance exercise resulted in a non-significant 3-fold increase in MuRF-1 mRNA expression (*P* = 0.10), which then fell just below baseline 24 h after the final training session (*P* = 0.15). There was a non-significant 1.4-fold increase in MAFbx mRNA expression following a bout of exercise in the untrained condition (*P* = 0.80), which then was seen to fall significantly below baseline after training (*P* = 0.01).

**Figure 7:**
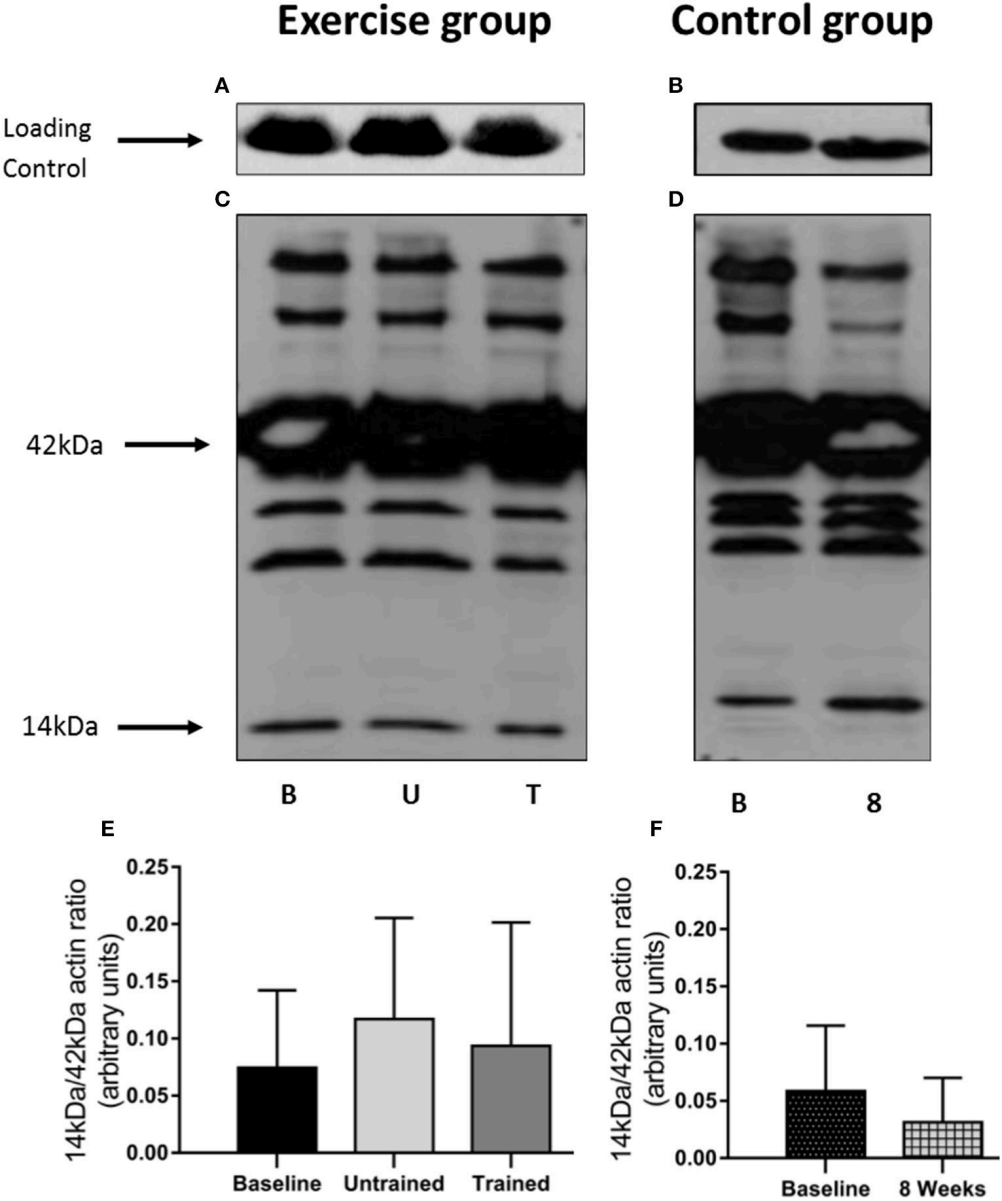
Abundance of the 14 kDa fragment in skeletal muscle biopsies in response to accustomed and unaccustomed resistance exercise. Samples from exercising CKD patients were drawn at baseline, 24 h after first bout of exercise (Untrained) and 24 h after final bout of exercise following 8 weeks resistance exercise training (Trained) and at baseline and 8 weeks later in the non-exercising control group. Representative blots are shown for loading controls in exercising **(A)** and control patients **(B)** and full experimental blots **(C,D)** that are labeled to show 42 kDa actin and 14 kDa actin fragment. Histograms **(E,F)** show densitometric data. Bm denotes Baseline; U, denotes untrained; T, denotes Trained; 8, denotes 8 weeks. Data are mean ± *SD*.

**Figure 8:**
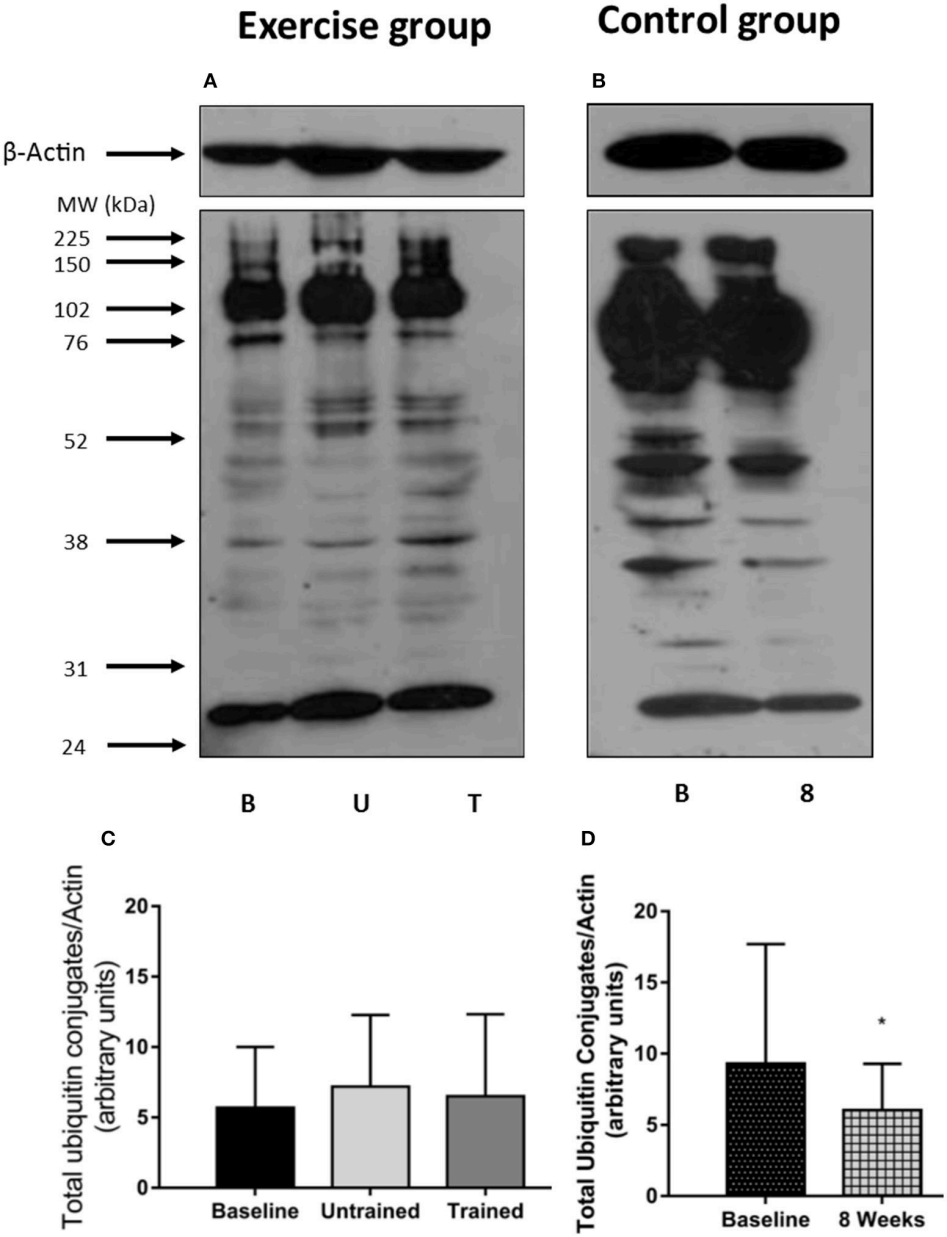
Abundance of ubiquitin conjugates in skeletal muscle biopsies in response to accustomed and unaccustomed resistance exercise. Samples from exercising CKD patients were drawn at baseline, 24 h after first bout of exercise (Untrained) and 24 h after final bout of exercise following 8 weeks resistance exercise training (Trained) and at baseline and 8 weeks later in the non-exercising control group. Representative ubiquitin-protein conjugates blots are shown for exercising **(A)** and control patients **(B)** together with β-Actin loading controls for these groups. Histograms show densitometric data **(C,D)**. B, denotes Baseline; U, denotes untrained; T, denotes Trained; 8, denotes 8 weeks. ^*^P < 0.05 vs. respective baseline sample. Data are mean ± *SD*.

The 14 kDa fragment is a cleavage product produced during the degradation of actin and myosin that is used as a biomarker of muscle proteolysis (Workeneh et al., 2006; Wang and Mitch, 2013). We found no evidence that unaccustomed or accustomed exercise resulted in any change in the amount of 14 kDa present in the insoluble fraction of the muscle biopsies (*P* = 0.36; Figure 7), or in the level of ubiquitin conjugation in response to either unaccustomed or accustomed exercise (*P* = 0.20; Figure 8), suggesting there was no change in the overall rate of protein degradation in these patients. Pairwise comparisons showed no change in the expression of MuRF-1 (*P* = 0.64), MAFbx (*P* = 0.62), or in the amount of the 14 kDa fragment (*P* = 0.65) in the control group. There was however, a significant decrease in the level of ubiquitin conjugates from baseline to 8 weeks (*P* = 0.02).

## Discussion

Evidence is accumulating for the benefits of exercise in advanced CKD making it a promising therapeutic intervention (Watson et al., 2014; Greenwood et al., 2015; Howden et al., 2015). However, little is known about the mechanisms of muscle wasting that is commonly seen in these patients, the ability of exercise to overcome it, or how these patients respond to exercise at a molecular level. There are a number of studies that describe the anabolic and inflammatory response to exercise in human CKD (Kouidi et al., 1998; Wagner et al., 2001; Kopple et al., 2007; Balakrishnan et al., 2010; Coletta et al., 2016), however to the authors acknowledge, this is the first study to describe this response to exercise training in non-dialysis CKD. Here, we show that when patients are untrained exercise results in a large increase in the expression of several inflammatory cytokines, and a suppression of IL-15. Exercise training however, does appear to reduce the expression of these inflammatory cytokines provoked by an acute bout of accustomed resistance exercise.

Inflammation seems to play a key role in the pathogenesis of muscle wasting in CKD (Wang and Mitch, 2014). Pro-inflammatory gene and protein expression are up-regulated in the skeletal muscle of patients with CKD (Garibotto et al., 2006; Verzola et al., 2011; Zhang et al., 2013), creating local and systemic inflammation that contributes to muscle atrophy through reduced protein synthesis and increased protein degradation. We, (Viana et al., 2014) and others, (Castaneda et al., 2004) have shown that both aerobic and resistance exercise may confer systemic anti-inflammatory benefits in pre-dialysis CKD, but little is known about the intramuscular inflammatory response to exercise in CKD. In healthy individuals, acute resistance exercise induces a transient inflammatory response that is required for appropriate muscle regeneration and adaptation (Louis et al., 2007; Pillon et al., 2013). We observed a previously undocumented inflammatory response to unaccustomed resistance exercise in the skeletal muscle of non-dialysis CKD patients, with significant increases in the expression of IL-6, MCP-1, and TNF-α. This large inflammatory response may have consequences for the activation of anabolic processes such as protein synthesis, as it is now established that there is a strong interaction between increased cytokine expression and suppression of insulin signaling. This likely occurs through activation of the suppressor of cytokine signaling (SOCS) proteins, which have been shown to degrade the IRS-1 protein (Kawazoe et al., 2001), or through activation of the NF-kβ pathway. The NF-kβ pathway also affects the abundance and activation of IRS-1 by increasing expression of a transmembrane glycoprotein SIRP-α (Thomas et al., 2013), a mechanism that is currently being explored as a contributor to muscle wasting in CKD. However, the magnitude of this inflammatory response was greatly reduced following 8 weeks of exercise training, suggesting that exercise training does not provoke a large and on-going inflammatory environment within skeletal muscle, which could be detrimental for muscle health. We also saw a suppression of IL-15 expression after unaccustomed exercise. IL-15 is known to have anabolic effects in skeletal muscle (Quinn et al., 2002) and its expression has been reported to increase 24 h after resistance exercise in healthy individuals (Nielsen et al., 2007). Regular resistance training appears to restore the IL-15 response, which may contribute to the significant muscle hypertrophy observed and previously reported (Watson et al., 2014).

Earlier work by Wang et al. (2009) has demonstrated that mice with CKD have blunted insulin/insulin like growth factor 1 (IGF-1) signaling which centers around a reduced phosphorylation of Akt in these animals and which ultimately results in muscle atrophy that is commonly seen in CKD. This reduction in Akt phosphorylation reduces the phosphorylation of the forkhead transcription factors (foxO) leading to an increase in the expression of the E3 ligases MuRF-1 and MAFbx and an acceleration of the ubiquitin proteasome system. On the other side of the equation, a reduction in Akt phosphorylation results in a suppression of protein synthesis. However, the authors were able to demonstrate that muscle overload in the CKD mice was able to restore Akt signaling, significantly elevating it above that in the non-exercised mice. This led to the conclusion that muscle overload can blunt the development of muscle atrophy in CKD, which was strongly linked to the observed increase in Akt phosphorylation. The results we present here support this previous observation in nephrectomised mice. In response to unaccustomed resistance exercise there was no change in Akt phosphorylation from baseline which was seen to significantly increase in response to resistance exercise following 8 weeks of training, where it was elevated 2-fold above baseline.

eEF2 is an important regulator of translation elongation, mediating translocation of the ribosome along the mRNA strand (Proud, 2015). Phosphorylation of eEF2 by eEF2 kinase prevents it from binding to the ribosome slowing the rate of elongation (Kenney et al., 2014). We found eEF2 phosphorylation was unchanged 24 h after a bout of resistance exercise both before and after training. There is little research on the effect of exercise on regulation of eEF2, but one study showed P-eEF2 was reduced 3 h following sprint exercise (Rundqvist et al., 2013). In the absence of direct protein turnover measurements we must interpret these results with caution—especially in light of an apparent disconnect between synthetic rates and phosphorylation of anabolic factors (Greenhaff et al., 2008). However, as there are now several reports that describe abnormal anabolic signaling in CKD (Chen et al., 2008; Wang et al., 2009), more detailed investigation into this area in response to exercise is warranted.

The myogenic regulatory factors (MRF's) MyoD and myogenin, are important regulators of muscle cell growth and differentiation and have an important role in the adaptive response to overload. It is generally accepted that an increase the expression of these proteins infers activation of myogenesis (Yang et al., 2005). Previous work in CKD mice has shown that the mRNA expression of these MRF's is reduced, but that muscle overload results in an increase in mRNA expression of both MyoD and Myogenin (Wang et al., 2009). Here we show that 24 h following an unaccustomed bout of resistance exercise there was no change in the mRNA expression of either MyoD or Myogenin. However, as no measure of muscle damage was made, it is difficult to appropriately interpret these results. We used a relatively moderate intensity resistance exercise protocol, which may not have caused any significant degree of muscle damage. If this is the case, there would not have been a sufficient stimulus to initiate the process of myogenesis. To draw firm conclusions, it would be important to investigate this further using a muscle damage protocol. A suppressed gene expression in response to exercise has been described previously in CKD and kidney transplant patients (Coletta et al., 2016), albeit in genes relating to the NFAT/calcineurin pathway, which taken together with the observations reported here, may suggest that exercise is not able to fully initiate molecular pathways necessary for adequate adaptation to exercise. This requires further investigation.

There is a consensus that skeletal muscle atrophy in CKD occurs largely through ubiquitin-proteasome mediated protein degradation (Wang and Mitch, 2013). Therefore, we investigated the effect of resistance exercise upon the two muscle specific E3 ligases, MuRF-1 and MAFbx, the level of total protein ubiquitin-conjugation and the concentration of the 14 kDa fragment present in the biopsies. We observed a 3-fold increase in MuRF-1 and a smaller 1.5-fold increase in MAFbx mRNA expression 24 h post exercise. Neither of which were significant, but these increases disappear after training where MAFbx expression was actually suppressed below baseline. This response is generally in line with that previously reported in healthy individuals where the increase in the mRNA message for these ligases tends to peak 1–4 h post exercise (Louis et al., 2007) and then falls below baseline. We believe that the small but non-significant increase in expression seen here to an unaccustomed and therefore potentially damaging bout of exercise, serves to remove and degrade damaged protein, which does not occur to the same extent following training (Constantin et al., 2013). This early increase in expression did not translate into a detectable increase in protein destruction as we did not find any changes in global ubiquitin conjugation, or in the amount of the 14 kDa fragment present. This suggests that resistance exercise does not negatively affect muscle protein balance, either acutely, or in the longer-term with training. Therefore, resistance exercise does not place an additional severe metabolic stress on the muscle of these patients, which could worsen existing muscle loss.

Myostatin is a potent negative regulator of growth (Han and Mitch, 2011). Following both unaccustomed and accustomed exercise we saw a significant reduction in myostatin expression that has been reported previously in healthy individuals (Louis et al., 2007) and is a normal response to exercise.

Finally, CKD patients experience high levels of oxidative stress, contributing to systemic inflammation, and ultimately muscle wasting (Sung et al., 2013). Depending upon its intensity, exercise can generate reactive oxygen species (ROS), which in healthy individuals increases the production of antioxidant enzymes (Gomez-Cabrera et al., 2008). If patients are unable to mount a suitable response to this transient increase in ROS production it may worsen oxidative stress in these patients. However, we did not see any evidence that oxidative stress was exacerbated by exercise. Whether, additional oxidative stress caused by the exercise was offset here by an increase in antioxidant enzyme production is unknown.

A limitation of this study concerns sampling time points. Post exercise biopsy samples were only collected 24 h after exercise and we therefore cannot comment on the intramuscular responses before 24 h. It is possible that other important effects on gene expression or phosphorylation events were missed and had returned to baseline by the time of the post exercise biopsy. The lack of a healthy control group here means we are unable to associate changes seen with disease specific mechanisms and to confirm that they do not simply reflect deconditioning. However, these responses in untrained healthy individuals are well documented and do appear to differ from the response we report in CKD patients. There are differences in eGFR at baseline between the two groups; there was however, no difference in the rate of change of eGFR over the course of the study. Finally, the markers we have chosen to study here are only static measures and provide no information on protein turnover kinetics, which can be studied using more invasive techniques.

In conclusion, our data has shown that CKD patients exhibit a large intramuscular inflammatory response to exercise in the untrained state. However, resistance exercise training attenuated the expression of these inflammatory markers following acute exercise, suggesting that this form of exercise does not provoke an on-going inflammatory response within the muscle. We have also shown that resistance exercise training was able to restore the expected increase in Akt phosphorylation in response to acute exercise, supporting the previous work by Wang et al. (2009). We believe that further investigation is warranted to better define the effect of exercise on the activation of proteins and inflammatory factors likely to be involved in a hypertrophic response in CKD.

## Author contributions

My co-authors have all contributed to this manuscript and relative contributions made by the listed authors are as follows; EW, JV, and AS were involved in the study design and developed the protocol and JB was responsible for patient recruitment. EW, NG, DW, and AS were involved in the sample collection and laboratory analysis. EW, DW, NM, JV, and AS were involved in data analysis and data interpretation. EW performed the literature search and compiled the figures and EW, JV, DW, NM, NG, JB, and AS were responsible for preparing the manuscript for submission. EW takes responsibility that this study has been reported honestly, accurately, and transparently; that no important aspects of the study have been omitted; and that any discrepancies from the study as planned have been explained. All authors approve this submission.

### Conflict of interest statement

The authors declare that the research was conducted in the absence of any commercial or financial relationships that could be construed as a potential conflict of interest.
