# Supplementary material for: The Effect of Resistance Exercise on Inflammatory and Myogenic Markers in Patients with Chronic Kidney Disease
Source: Front Physiol. 2017 Jul 28;8:541. doi: 10.3389/fphys.2017.00541 (PMC5532513; doi:10.3389/fphys.2017.00541)
Supplement: Supplementary file 1 [file DataSheet1.docx]

Supplementary methods

Validation of housekeeping gene

A complete series of samples from one participant that was randomised to the exercise group was run on an endogenous control plate (Applied Biosystems 4396840) which allows for the determination of the stability of 32 commonly used housekeeping genes over three experimental conditions (e.g. Baseline, Untrained, Trained; Figure 1). Based upon expression level and %CV across the three sampling times used here, GAPDH, TBP and HMBS were chosen for further validation. We tested the stability of these three genes in a further 5 patients and noted %CVs of 2.4% 4.0% and 6.2% for TBP, HMBS and GAPDH respectively. Therefore TBP was chosen as an internal control for this analysis. TBP %CV over all patients analysed in the study = 2.2%

Figure 1. Results of endogenous control plate. Bars show % CV over the three biopsy samples in a single exercising patient.
